# Cervical Myofascial Pain Is Associated with an Imbalance of Masticatory Muscle Activity

**DOI:** 10.3390/ijerph19031577

**Published:** 2022-01-29

**Authors:** Michał Ginszt, Jacek Szkutnik, Grzegorz Zieliński, Magdalena Bakalczuk, Małgorzata Stodółkiewicz, Monika Litko-Rola, Apolinary Ginszt, Mansur Rahnama, Piotr Majcher

**Affiliations:** 1 Department of Rehabilitation and Physiotherapy, Medical University of Lublin, 20-093 Lublin, Poland; apolinary.ginszt@umlub.pl (A.G.); piotr.majcher@umlub.pl (P.M.); 2 Independent Unit of Functional Masticatory Disorders, Medical University of Lublin, 20-093 Lublin, Poland; jacek.szkutnik@umlub.pl (J.S.); magdalena.bakalczuk@umlub.pl (M.B.); malgorzata.stodolkiewicz@umlub.pl (M.S.); monika.litko@umlub.pl (M.L.-R.); 3 Department of Sports Medicine, Medical University of Lublin, 20-093 Lublin, Poland; grzegorz.zielinski@umlub.pl; 4 Department of Oral Surgery, Medical University of Lublin, 20-093 Lublin, Poland; mansur.rahnama@umlub.pl

**Keywords:** masticatory muscles, neck muscles, electromyography, myofascial pain, balance

## Abstract

This study aimed to assess the relationship between the occurrence of cervical myofascial pain with active myofascial trigger points (MTrPs) within the upper trapezius muscle and the electromyographic asymmetry index (AsI) of masticatory muscles: temporalis anterior (TA), superficial part of the masseter muscle (MM), and anterior belly of the digastric muscle (DA). The study group comprised 100 subjects (80 women and 20 men) aged 18 to 30 years (mean 23 ± 2.6 years) reporting pain in the neck muscles, diagnosed with myofascial pain with active MTrPs only within the upper trapezius muscle. The control group comprised 60 healthy, pain-free subjects (42 women and 18 men) aged 20 to 30 years (mean 22.8 ± 2.6 years) without MTrPs in the upper trapezius muscle. The palpation measurement, based on the diagnostic criteria of Travell and Simons, was used to diagnose active MTrPs. The masticatory muscle activity was recorded using an 8-channel device for surface electromyography—BioEMG III^TM^. Significant differences in electromyographic patterns between the group with MTrPs in the right side of upper trapezius muscle and the control group were observed within resting activity for the AsI TA (MTrPs: 8.64 vs. controls: −3.22; *p* = 0.001) and AsI MM (MTrPs: 7.05 vs. controls: −2.09; *p* = 0.018). Controls presented different electromyographic patterns during maximum voluntary clenching with cotton rolls between teeth within masseter muscle compared to the MTrPs group (MTrPs: 9.27 vs. controls: −0.43 vs. *p* = 0.041). Participants with MTrPs in the left side of upper trapezius muscle presented predomination of left-sided electromyographic patterns at rest within temporalis anterior in comparison to controls (MTrPs: −19.22 vs. controls: −3.22; *p* = 0.001). MTrPs within the trapezius muscle may be related to asymmetry within the masticatory muscle activity, suggesting that the presence of myofascial pain within the cervical muscles plays a role in the imbalance of the stomatognathic system. A unilateral active MTrPs within the trapezius muscle may increase the sEMG activity on the same side of the temporalis anterior and masseter muscles.

## 1. Introduction

Myofascial pain is defined as a musculoskeletal disorder that causes pain in the area of a muscle and fascia. The pain can be characterized by multiple myofascial trigger points (MTrPs), which are defined as exquisitely tender spots in taut bands of a muscle or fascia that produce local and referred pain [1,2]. Trigger points are classified mainly as being active or latent [3]. Moreover, latent MTrPs may be converted to active MTrPs by continuous mechanical or chemical detrimental stimuli [4,5]. The most common causes of MTrPs are direct trauma, sudden overstretching of the muscle or chronic injuries, and overload of the muscular system. The potential mechanism is local ischemia, which leads to a lowered pH and a subsequent release of several inflammatory mediators in muscle tissue [1]. Both active and latent MTrPs are associated with dysfunction of the myofascial system, muscle weakness, reduced range of motion, and symptoms related to the autonomic nervous system, such as excessive sweating, tearing, and changes in skin temperature. However, in the case of latent MTrPs, clinical manifestation is noticeably lower [6,7]. Besides, active MTrPs are associated with spontaneous pain in the surrounding tissue or distant sites by referred pain phenomenon, compared to latent MTrPs, which are not associated with a spontaneous pain complaint [8]. It is worth noting that both active and latent MTrPs are also accompanied by changes in muscle activity, not only within the muscle with MTrPs but also in antagonistic muscles [9,10]. The presence of MTrPs can be manifested as an increase in the resting muscle activity and a decrease in the amplitude of the electromyographic signal during muscle contraction [11]. Although the literature about MTrPs prevalence is sparse due there being only a few studies, with very low sample sizes and design limitations, MTrPs are very common [1,12]. According to Travell and Simons’ diagnostic criteria, the current gold standard for the MTrPs diagnosis is the manual examination via palpation (e.g., taut band, tender nodule on the taut band with increased pain on pressure, referred recognition pain, and local twitch response) [8]. According to an up-to-date systematic review, spot tenderness (hypersensitive spot/nodule, taut band, or tender spot in a taut band), referred pain, and local twitch response were the three most common criteria [13].

The phenomenon of referred pain associated with myofascial pain and MTrPs over a long period is a topic of discussion concerning craniofacial pain. However, the mechanisms causing this phenomenon have not been unequivocally explained and scientifically proven [8]. A possible explanation for referred pain is increased synaptic efficiency by activating ineffective synapses at the dorsal horn due to central sensitization. Active MTrPs stimulate muscle nociceptors that, upon sustained noxious stimulation, initiate motor and sensory changes in the peripheral and central nervous systems [14]. Referred pain from cervical muscles, e.g., upper trapezius, spreads to the head, simulating symptoms patients perceive with temporomandibular disorders (TMDs) [15]. More precisely, active MTrPs in the upper trapezius muscle may be responsible for developing masticatory muscle pain through the mechanism of referred pain. Moreover, active MTrPs in the upper trapezius muscle are also related to tension-type headache (TTH) episodes [16]. Electromyographic studies have also demonstrated the altered distribution of masticatory muscle activity in individuals with neck pain [17,18].

Masticatory function requires synergistic interactions between jaw bones, temporomandibular joints, teeth, and masticatory muscles [19]. Several studies reported that imbalanced masticatory muscle activity is observed in subjects with pathological or abnormal conditions (e.g., TMDs, malocclusion) [20,21,22]. To indicate the balance within electromyographic activity of the right and left side of masticatory muscles, the asymmetry index (AsI) was developed [23]. The surface electromyography (sEMG) symmetry ratio between the right and left side may suggest structural or functional disorders within the stomatognathic system. To date, several studies have used AsI to assess masticatory muscle activity in individuals with clinical symptoms of mandibular disorders, different occlusal and skeletal classes, during orthodontic treatments, and in the healthy population [24,25,26]. A thorough understanding of the mechanisms of referred pain induced in masticatory muscles from active myofascial trigger points in muscles of the cervical spine may be important in a more accurate understanding of the etiology and development disorders such as tension-type headaches and masticatory muscle pain. Therefore, this study aimed to assess the relationship between the occurrence of myofascial pain with active myofascial trigger points within the upper trapezius muscle and the asymmetry index of masticatory muscles. The null hypothesis is that the presence of myofascial pain with MTrPs within the upper trapezius muscle is related to the imbalance of masticatory muscle activity.

## 2. Materials and Methods

### 2.1. Study Population

This study was carried out at the Department of Functional Masticatory Disorders, Medical University of Lublin, by experienced dentists and physiotherapists. The tests were carried out according to the Helsinki Declaration’s recommendations and with the Bioethics Committee’s consent of the Medical University of Lublin (KE-0254/346/2016). All participants were informed about the aim of the study and have given written permission for the research. 

A total of 200 adults between the ages of 18–30 were recruited to the study. During qualifying for the research, all participants were clinically examined based on a two-axis Research Diagnostic Criteria for Temporomandibular Disorders (RDC/TMD) by experienced dentists specializing in dental prosthetics (author J.S.). The measurements obtained in the RDC/TMD examination were used to assess the range of active maximum mouth opening. Based on Travel and Simons’ diagnostic criteria, the flat palpation technique was used to diagnose myofascial pain with active MTrPs within the upper trapezius muscle by an experienced physiotherapist specializing in myofascial pain syndrome diagnosis and management (author M.G.) [27]. The following criteria for active MTrPs were used in the present study: the presence of a taut band within the upper trapezius muscle; the presence of a spot tenderness within the taut band; the reproduction of pain complaints during mechanical stimulation of the spot tenderness; and the reproduction of a referred pain with mechanical stimulation of the spot tenderness, according to the protocol of Barbero et al. [28]. The inclusion criteria used in the study were: age range 18–30 years; good or very good general health status according to the RDC/TMD questionnaire; the presence of myofascial pain with active MTrPs in the upper trapezius muscle according to diagnostic criteria presented by Simons et al. (study group); and absence of active and/or latent MTrPs in the upper trapezius muscle (controls). The following exclusion criteria were used: the occurrence of headache and cervical spine pain within the month preceding the examination (*n* = 8); head and neck injuries within the last 6 months before the study; previous head and neck surgical treatment within the last 6 months before the examination; pregnancy (*n* = 4); craniofacial trauma; class II and III of the bite according to Angle’s classification (*n* = 10); open bite (*n* = 3); lack of four support zones in dental arches (*n* = 5); lack of more than four teeth within both dental arches; carious or damaged dental tissues (*n* = 4); any periodontal pathology; any pathology or asymmetry in craniofacial structures; any form of TMDs found according to the RDC/TMD (*n* = 6); condition during orthodontic treatment; possession of dental prostheses (regardless of type); Botox therapy; and mental and neurological disorders.

After applying the above criteria, 100 subjects (80 women and 20 men) aged from 18 to 30 years (mean 23 ± 2.6 years), reported pain in the neck muscles and those diagnosed with myofascial pain with active MTrPs only in the upper trapezius muscle were qualified to the study group. The presence of active MTrPs in the upper trapezius muscle was found unilaterally (right side: *n* = 30; left side: *n* = 48) or bilaterally (*n* = 22). The control group comprised 60 healthy pain-free subjects (42 women and 18 men) aged from 20 to 30 years (mean 22.8 ± 2.6 years) who met the criteria for the study group and without MTrPs in the upper trapezius muscle.

### 2.2. Electromyographic Measurements

**Figure 1:**
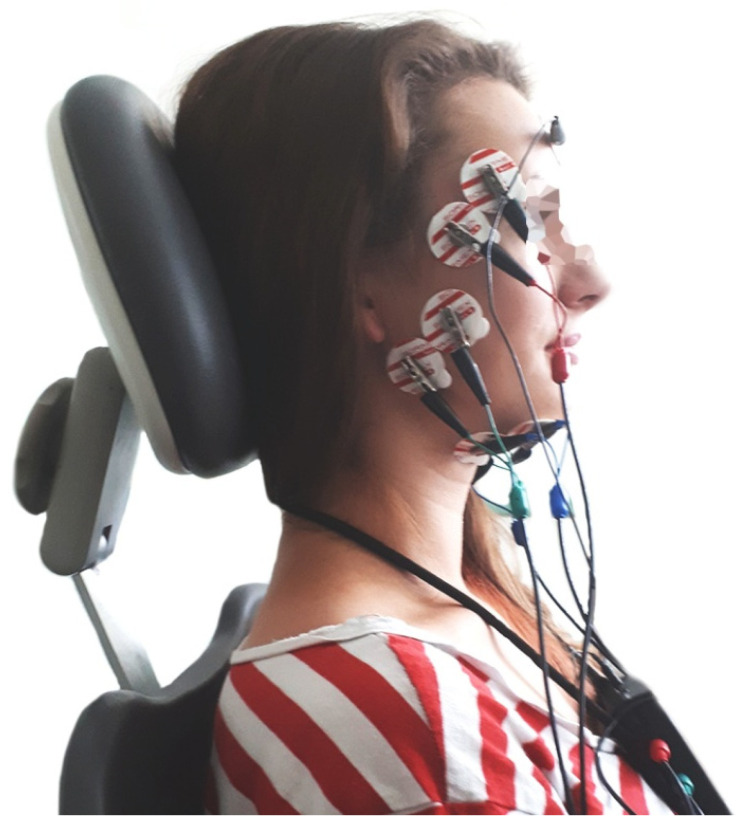
sEMG electrodes placement during the electromyographic examination.

The electromyographic examination was carried out in a dental chair in a sitting position, with the body perpendicular to the ground, the head resting on the chair’s headrest, and the lower limbs upright and arranged parallel to each other. The height of the headrest was adjusted individually to set the head, neck, and torso of the subjects in a straight line. The sEMG examinations were conducted between 8 and 12 a.m. to minimize the influence of daily fluctuations of muscle activity. The muscle activity was recorded using an 8-channel device for surface electromyography—BioEMG III^TM^ (BioResearch Associates, Inc., Milwaukee, WI, USA). Electromyographic signals were obtained from six channels. Microvolt signals were amplified with minimal noise to 5000 times their original levels. The noise was reduced by 40 dB using the Noise Buster digital filtering in the BioPAK Measurement System (BioResearch Associates, Inc., Milwaukee, WI, USA), which automatically removes 99% of any remaining 50/60 Hz noise. Before placing the surface electrodes, the skin was cleaned with 90% ethanol solution to reduce electrode–skin impedance. Next, surface electrodes (Ag/AgCl with a diameter of 30 mm and a conductive surface of 16 mm (SORIMEX, Toruń, Poland) were placed following the course of the muscle fibers of the temporalis anterior (TA), the superficial masseter muscle (MM), and anterior bellies of the digastric muscle (DA), according to the SENIAM (Surface EMG for Non-Invasive Assessment of Muscles) guidelines [29]. The arrangement of the electrodes symmetrically on the skin covering the examined muscles on both sides following the course of muscle fibers was preceded by palpation of the muscles during mandibular movements, according to the placement technique described by Ferrario and Sforza [30]. The electrodes on the superficial masseter muscle were located along the line from the mandible angle to the inferior border of the zygomatic bone. The electrodes on the anterior part of the temporal muscle were arranged along a perpendicular line from the superior border of the zygomatic bone to a cranial bone (in the projection of the sphenoid bone). The electrodes on the anterior bellies of the digastric muscle were placed approximately 1 cm medial to the base of the mandible. The edges of the electrodes covering the skin above an examined muscle were in contact to maintain a constant spacing between the electrodes. The reference electrode was placed on the forehead, in the center of the frontal bone (Figure 1).

Muscle activity was assessed during resting mandibular position (10 s), during clenching in intercuspal position (three times for 3 s each, with 2 s of rest between), during clenching on cotton rolls between teeth (three times for 3 s each, with 2 s of rest between) and during active maximum mouth opening (three times for 3 s each, with 2 s of rest between). The asymmetry index (AsI) was used to indicate the relative contributions of the right and left side within temporalis anterior (AsI TA), masseter muscle (AsI MM), and digastric muscle (AsI DA) using the following equations:

AsI TA = (RMS TA right − RMS TA left)/(RMS TA right + RMS TA left) × 100

AsI MM = (RMS MM right − RMS MM left)/(RMS MM right + RMS MM left) × 100

AsI DA = (RMS DA right − RMS DA left)/(RMS DA right + RMS DA left) × 100

The asymmetry index varies between −100 and +100. The negative (−) values indicate the predominance of the left muscle activity. The positive (+) values suggest muscle advantage within the right side of the craniofacial complex. Both indices reaching values close to 0 indicate the symmetrical and equal bioelectric involvement of the masseter, temporalis anterior, and digastric muscle, according to Naeije et al. and Ferrairo et al. protocols [23,31].

### 2.3. Statistical Analysis

The repeatability of the sEMG protocol was tested by duplicate sEMG measurements on the 10 participants. The two independent sEMG measurements were separated by 5 min rest between activities. There were no significant differences (*p* > 0.05) between repeated sEMG records in all analyzed variables (resting mandibular position, maximum voluntary clenching, maximum voluntary clenching on cotton rolls between teeth, maximum mouth opening). The checklist developed by the Strengthening the Reporting of Observational Studies in Epidemiology (STROBE) initiative was used to assess the methodological quality of the presented study [32]. The data comparison was performed using the IBM SPSS STATISTICS 21 program (IBM Corp., Armonk, NY, USA). Pearson’s Chi-square test was used to check the gender equality between the study group and controls. The normality of the distribution of variables was verified using the Shapiro–Wilk test and the Kolmogorov–Smirnov test (with the Lillierfors correction). All distributions did not fulfill normal distribution, which is why the non-parametric Mann–Whitney U test was used later. Effect sizes were determined using the Cohen d method and interpreted as small (0.2), medium (0.5), and large (0.8) effect sizes. The differences were considered statistically significant if the level of test probability was lower than the assumed level of significance (*p* < 0.05).

## 3. Results

Statistical analysis showed that there was no significant difference between the group with active MTrPs in the upper trapezius muscle and the control group in terms of age (*p* = 0.235), maximum mouth opening (MMO) values (*p* = 0.263), and gender (*p* = 0.053) (Table 1).

There were no significant differences between all participants with MTrPs in the upper trapezius muscle and the control group during the resting mandibular position, maximum voluntary clenching in the intercuspal position, maximum voluntary clenching on cotton rolls between teeth, and during maximum active mouth opening (Table 2).

Significant differences in electromyographic patterns between the group with MTrPs in the right side of the upper trapezius muscle and the control group were observed within resting activity for the AsI TA (MTrPs: 8.64 vs. controls: −3.22; *p* = 0.001) and AsI MM (MTrPs: 7.05 vs. controls: −2.09; *p* = 0.018). In addition, controls presented different electromyographic patterns during maximum voluntary clenching with cotton rolls between teeth within the masseter muscle compared to the MTrPs group (MTrPs: 9.27 vs. controls: −0.43 vs. *p* = 0.041). The abovementioned indices showed a predominance of resting and functional masticatory muscle activity on the right side of the craniofacial complex within the MTrPs group. Regarding other AsI indices, the differences between the studied groups did not reach the assumed significance level (Table 3).

Table 4 demonstrates the differences within resting AsI TA between the group with MTrPs in the left side of the upper trapezius muscle and the control group. People with MTrPs presented predomination of left-sided electromyographic patterns at rest within temporalis anterior in comparison to controls (MTrPs: −19.22 vs. controls: −3.22; *p* = 0.001) (Table 4).

The comparison of mean AsI between the group with MTrPs in both sides of the upper trapezius muscle and the control group showed significant differences only within AsI DA during maximum active mouth opening (MTrPs: 12.32 vs. controls: 2.82; *p* = 0.041), demonstrating digastric muscle advantage within the right side of the craniofacial complex during functional activity (Table 5).

## 4. Discussion

The hypothesis that myofascial pain with active MTrPs within the upper trapezius is related to the imbalance among masticatory muscle activity seems to be confirmed in the presented research. Significant differences in the mean value of the asymmetry index of the temporalis anterior during the resting activity were observed in the group with active MTrPs on the right trapezius muscle and among participants with active MTrPs on the left part muscle compared to controls. Moreover, significant differences in masseter muscle symmetry were observed between the group with active MTrPs on the right part of the trapezius muscle and controls during maximum voluntary clenching with cotton rolls between teeth. However, there were no significant differences in the asymmetry index between all patients with MTrPs and the control group. The only difference was noticed within digastric muscle asymmetry during maximum active mouth opening between the group with MTrPs in both sides of the upper trapezius muscle and the controls, demonstrating digastric muscle advantage within the right-side craniofacial complex during functional activity. The lack of significant differences may be due to increased resting activity of the temporalis anterior, depending on the side on which MTrPs are located within the trapezius muscle. Therefore, the asymmetry index score for all patients with MTrPs could be biased by the above electromyographic variables. Moreover, the lack of differences may be related to the steady increase in the resting activity of the masticatory muscles within both sides, which could be influenced by the bilateral presence of MTrPs in the descending part of the trapezius muscle.

Our results show that there may be a relationship between the occurrence of MTrPs within the upper trapezius and changes in the resting activity of the masticatory muscles. However, the relation mentioned above may only slightly concern the functional activity of the stomatognathic system. In numerous studies, disturbances in the masticatory symmetry were connected to pain in the head area and other symptoms related to TMDs and masticatory muscle pain [33,34,35,36]. Moreover, active MTrPs in the cervical musculature may be responsible for headache reproduction in women with migraines [37]. On the other hand, incorrect recruitment of the trapezius muscle via MTrPs during clenching and jaw movements can also alter the activation patterns of the masticatory muscles [38,39,40].

Despite many scientific studies and clinical observations, the relationship between the cervical spine and the stomatognathic system is still not fully understood. Confirmation of the theory and mechanisms of the transferred pain formation within masticatory muscles from active MTrPs in the cervical spine muscles is still a challenge for modern medicine. Mandibular and neck activities are interrelated, and alterations in one part can also derange the other one. Moreover, the trapezius muscle is partly innervated by the XI cranial nerve, whose motor nucleus is reached by the neural connections of the brain stem [41]. The peripheral nociception may influence central sensitization mechanisms, which increases the sensitivity to peripheral pain [42]. Thus, prolonged nociceptive information from MTrPs within the trapezius muscle may lead to pain and disturbing functional activity within the stomatognathic system. In addition, the referred pain from the upper trapezius simulates symptoms perceived by patients with TMDs and shows some similarities in the patterns of electromyographic activity [15,38]. Hence, the phenomenon of transferred pain and a change in masticatory muscle activity indicate the possibility of considering the myofascial disorders of the cervical spine muscles as predictors of the TTH and TMDs. However, so far, there is no clear evidence supporting the above assumptions.

Our findings add new insights to the debate about the underlying cause of imbalance of masticatory muscle activity. The Orofacial Pain Prospective Evaluation and Risk Assessment Study (OPPERA), showed a significantly greater number of painful areas in the neck muscles in people with TMDs than in control groups [43]. Pain in the neck area was also shown to be twice as frequent in children diagnosed with TMDs compared with the control group [44]. Based on the results of electromyographic examinations, an increase in the resting activity of the trapezius muscle was also observed in patients with masticatory muscle pain and TMDs compared to controls without the painful form of TMDs. The observations mentioned above indicate the presence of a functional connection between the muscles of the cervical spine and the stomatognathic system [45,46]. In addition, altered distribution of the masticatory muscle activity was observed in patients with cervical spine pain [17,18]. Some previous studies have shown that whiplash injuries can alter the motor control of the masticatory muscles and the movement pattern during mouth opening [47,48,49]. Moreover, it has been shown that the changes in activity of the masseter muscles may depend on the cervical spine’s position during the maximum clenching of the teeth in healthy individuals [50]. In addition, increasing pain in the cervical spine may reduce the pressure pain threshold within the masticatory muscles [51]. Hence, based on the present study and the abovementioned scientific reports, the presence of MTrPs in the trapezius muscle may be considered a factor predisposing to muscular imbalance and pain in the craniofacial structures related to TMDs. However, the mechanisms causing this phenomenon have not been fully explained and should be confirmed in future studies.

A practical implication of this study is that MTrPs in the trapezius muscle may cause asymmetry in the masticatory muscle activity. Hence, diagnosis of MTrPs within the trapezius muscle should be performed when changes in the masticatory muscle activity are observed. Early initiation of trapezius trigger point therapy may prevent the development of dysfunction within the stomatognathic system. Thus, future clinical trials are needed to confirm the effectiveness of trigger point therapy application within trapezius muscle concerning masticatory muscles.

The presented study has several limitations. Firstly, the diagnostic criteria for TMDs were replaced by DC/TMDs in 2014. However, in this study, the previous version was used. There is no validated Polish version of the DC/TMDs so far. Therefore, the RDC/TMDs were used. Secondly, based on Travel and Simons’ diagnostic criteria, the flat palpation technique was used to diagnose MTrPs within the upper trapezius muscle. Even though elastography, Doppler imaging, diagnostic ultrasound, or biomarkers can be used in the diagnosis of MTrPs, we decided to use the current gold standard for the MTrPs diagnosis via palpation. Thirdly, the study sample consists of young adults aged from 18 to 30 years. We decided to include only young adults in the presented research to minimize the influence of age on the study results. Thus, future studies should consist of an expanded age range population.

## 5. Conclusions

MTrPs within the trapezius muscle may be related to asymmetry within the masticatory muscle activity, suggesting the presence of myofascial pain within trapezius muscle plays a role in the imbalance of the stomatognathic system. A unilateral active MTrPs within the trapezius muscle may increase the sEMG activity on the same side of the temporalis anterior and masseter muscles.

## Figures and Tables

**Table 1:** The comparison of average age, mean maximum mouth opening (MMO), and gender between the group with active myofascial trigger points (MTrPs) in the upper trapezius muscle and control group.

	MTrPs Group *n* = 100		Control Group *n* = 60		Z	*p*
	M	SD	M	SD		
Mean Age (years)	23.04	2.56	22.75	2.58	1.186	0.235
Mean MMO (mm)	50.29	6.88	51.57	6.10	−1.120	0.263
Sex	Women (*n*)	Men (*n*)	Women (*n*)	Men (*n*)	χ2	*p*
	80	20	42	18	3.75	0.053

**Table 2:** The comparison of mean asymmetry index (AsI) between the group with MTrPs in the upper trapezius muscle and the control group.

		MTrPs Group *n* = 100		Control Group *n* = 60		Z	*p*
		M	SD	M	SD		
Resting activity	AsI TA	−7.42	21.47	−3.22	16.46	−1.107	0.268
	AsI MM	1.75	20.20	−2.09	16.27	−1.378	0.168
	AsI DA	0.38	11.45	−0.52	13.12	−0.240	0.811
Maximum voluntary clenching in intercuspal position	AsI TA	−5.51	21.45	−4.47	15.65	−0.864	0.388
	AsI MM	3.47	24.20	−1.31	21.33	−1.079	0.281
	AsI DA	−0.40	20.72	−0.20	15.13	−0.143	0.886
Maximum voluntary clenching with cotton rolls between teeth	AsI TA	−5.78	16.19	−3.31	15.18	−1.227	0.220
	AsI MM	4.28	21.27	−0.43	16.93	−1.688	0.091
	AsI DA	0.96	19.66	2.90	18.49	−0.063	0.949
Maximum active mouth opening	AsI TA	−1.39	17.72	−0.38	18.97	−0.389	0.697
	AsI MM	2.34	20.05	1.27	19.56	−0.226	0.822
	AsI DA	3.57	16.02	2.82	19.53	−0.044	0.965

MTrPs—Myofascial trigger points; AsI TA—Asymmetry index for temporalis anterior; AsI MM—Asymmetry index for masseter muscle; AsI DA—Asymmetry index for digastric muscle.

**Table 3:** The comparison of mean asymmetry index (AsI) between the group with MTrPs in the right side of the upper trapezius muscle and the control group.

		MTrPs Group *n* = 30		Control Group *n* = 60		Z	*p*
		M	SD	M	SD		
Resting activity	AsI TA	8.64	17.46	−3.22	16.46	−3.381	0.001 * ES = 0.66
	AsI MM	7.05	17.59	−2.09	16.27	−2.371	0.018 * ES = 0.66
	AsI DA	3.98	8.46	−0.52	13.12	−1.447	0.148
Maximum voluntary clenching in intercuspal position	AsI TA	−3.56	17.63	−4.47	15.65	−0.009	0.993
	AsI MM	7.77	26.55	−1.31	21.33	−1.498	0.134
	AsI DA	4.75	23.30	−0.20	15.13	−1.695	0.090
Maximum voluntary clenching with cotton rolls between teeth	AsI TA	−5.91	14.93	−3.31	15.18	−1.224	0.221
	AsI MM	9.27	22.81	−0.43	16.93	−2.046	0.041 * ES = 0.66
	AsI DA	2.87	23.23	2.90	18.49	−0.556	0.578
Maximum active mouth opening	AsI TA	−1.86	13.04	−0.38	18.97	−0.330	0.742
	AsI MM	3.09	20.99	1.27	19.56	−0.184	0.854
	AsI DA	−0.66	15.54	2.82	19.53	−0.937	0.349

MTrPs—Myofascial trigger points; AsI TA—Asymmetry index for temporalis anterior; AsI MM—Asymmetry index for masseter muscle; AsI DA—Asymmetry index for digastric muscle; ES—Effect size; * Significant difference.

**Table 4:** The comparison of mean asymmetry index (AsI) between the group with MTrPs in the left side of the upper trapezius muscle and the control group.

		MTrPs Group *n* = 48		Control Group *n* = 60		Z	*p*
		M	SD	M	SD		
Resting activity	AsI TA	−19.22	19.47	−3.22	16.46	−4.408	0.001 * ES = 0.74
	AsI MM	1.27	19.77	−2.09	16.27	−1.076	0.282
	AsI DA	−0.82	12.28	−0.52	13.12	−0.185	0.853
Maximum voluntary clenching in intercuspal position	AsI TA	−8.96	24.93	−4.47	15.65	−1.935	0.053
	AsI MM	0.32	20.74	−1.31	21.33	−0.427	0.670
	AsI DA	−1.72	17.64	−0.20	15.13	−0.532	0.595
Maximum voluntary clenching with cotton rolls between teeth	AsI TA	−6.90	17.13	−3.31	15.18	−1.305	0.192
	AsI MM	−0.99	17.72	−0.43	16.93	−0.476	0.634
	AsI DA	0.62	17.64	2.90	18.49	−0.396	0.692
Maximum active mouth opening	AsI TA	−1.31	20.85	−0.38	18.97	−0.581	0.561
	AsI MM	2.53	17.75	1.27	19.56	−0.077	0.938
	AsI DA	2.21	16.19	2.82	19.53	−0.606	0.545

MTrPs—Myofascial trigger points; AsI TA—Asymmetry index for temporalis anterior; AsI MM—Asymmetry index for masseter muscle; AsI DA—Asymmetry index for digastric muscle; ES—Effect size; * Significant difference.

**Table 5:** The comparison of mean asymmetry index (AsI) between the group with MTrPs in both sides of the upper trapezius muscle and the control group.

		MTrPs Group *n* = 22		Control Group *n* = 60		Z	*p*
		M	SD	M	SD		
Resting activity	AsI TA	−3.57	14.21	−3.22	16.46	−0.042	0.967
	AsI MM	−4.42	23.27	−2.09	16.27	−0.628	0.530
	AsI DA	−1.90	12.42	−0.52	13.12	−0.743	0.457
Maximum voluntary clenching in intercuspal position	AsI TA	−0.64	17.06	−4.47	15.65	−0.722	0.470
	AsI MM	4.50	27.85	−1.31	21.33	−0.649	0.516
	AsI DA	−4.53	22.76	−0.20	15.13	−0.748	0.454
Maximum voluntary clenching with cotton rolls between teeth	AsI TA	−3.14	16.18	−3.31	15.18	−0.063	0.950
	AsI MM	8.99	24.29	−0.43	16.93	−1.706	0.088
	AsI DA	−0.90	19.29	2.90	18.49	−0.199	0.842
Maximum active mouth opening	AsI TA	−0.93	16.49	−0.38	18.97	−0.230	0.818
	AsI MM	0.91	24.04	1.27	19.56	−0.314	0.754
	AsI DA	12.32	13.42	2.82	19.53	−2.041	0.041 * ES = 0.59

MTrPs—Myofascial trigger points; AsI TA—Asymmetry index for temporalis anterior; AsI MM—Asymmetry index for masseter muscle; AsI DA—Asymmetry index for digastric muscle; ES—Effect size; * Significant difference.

## Data Availability

The data presented in this study are available on request from the corresponding author.
